# New reports of parasitism by *Synhimantus* (*Dispharynx*) *nasuta* (Rudolphi, 1819) (Nematoda: Acuariidae) in wild birds in Brazil

**DOI:** 10.1590/S1984-29612023033

**Published:** 2023-06-23

**Authors:** Allan de Jesus Mendonça Severino, Alex Júnior Rocha, Fabiano Matos Vieira, Luís Cláudio Muniz-Pereira, Sueli de Souza Lima

**Affiliations:** 1 Laboratório de Helmintoses Intestinais, Departamento de Parasitologia, Universidade Federal de Minas Gerais – UFMG, Campus Pampulha, Belo Horizonte, MG, Brasil; 2 Laboratório de Taxonomia e Ecologia de Helmintos Odile Bain, Departamento de Zoologia, Universidade Federal de Juiz de Fora – UFJF, Campus Universitário, Juiz de Fora, MG, Brasil; 3 Universidade Federal do Vale do São Francisco – UNIVASF, Petrolina, PE, Brasil; 4 Laboratório de Helmintos Parasitos de Vertebrados, Instituto Oswaldo Cruz – IOC, Fundação Oswaldo Cruz – FIOCRUZ, Rio de Janeiro, RJ, Brasil

**Keywords:** Nematoda, wild birds, morphology, Zona da Mata, new records, Nematoda, aves silvestres, morfologia, Zona da Mata, novos registros

## Abstract

The aim of this study was to register the first occurrence of *Synhimantus* (*Dispharynx*) *nasuta* (Nematoda: Acuariidae) in some species of wild birds in Brazil. In addition, the aim was to deepen the knowledge about the morphology of this species through analysis using scanning electron microscopy. Nematodes were collected in wild birds of the species *Turdus leucomelas*, *T. rufiventris*, *Mimus saturninus*, *Pitangus sulphuratus, Megascops choliba*, *Tyto furcata*, and *Falco sparverius*. The morphological and morphometric data observed in the nematodes prove that these parasites are *S*. (*D.*) *nasuta*. This study also provides morphological data from light microscopy and scanning electron microscopy (SEM), as well as the morphometry of this nematode in each host species. Therefore, the current study confirms the first record of this nematode in *F*. *sparverius* and *T*. *furcata* in South America and, at the same time, these findings expand the host range of this parasite species worldwide, through the first records in *M*. *choliba*, *M*. *saturninus*, *T*. *leucomelas* and *T*. *rufiventris*.

## Resumo

O objetivo deste estudo foi registrar a primeira ocorrência de *Synhimantus* (*Dispharynx*) *nasuta* (Nematoda: Acuariidae) em algumas espécies de aves silvestres do Brasil. Além disso, pretendeu-se aprofundar o conhecimento sobre a morfologia desta espécie por meio de análises por microscopia eletrônica de varredura. Os nematoides foram coletados em aves silvestres das espécies *Turdus leucomelas*, *T. rufiventris*, *Mimus saturninus*, *Pitangus sulphuratus*, *Megascops choliba*, *Tyto furcata* e *Falco sparverius*. Os dados morfológicos e morfométricos observados nos nematoides comprovaram que esses parasitos são *S.* (*D.*) *nasuta*. Este estudo também fornece dados morfológicos de microscopia de luz e microscopia eletrônica de varredura (MEV), assim como a morfometria deste nematoide em cada espécie de hospedeiro. Portanto, o presente estudo confirma o primeiro registro desse nematoide em *F. sparverius* e *T. furcata* na América do Sul e, ao mesmo tempo, esses achados ampliam a gama de hospedeiros dessa espécie de parasito em todo o mundo, por meio dos primeiros registros em *M. choliba*, *M. saturninus*, *T. leucomelas* e *T. rufiventris*.

## Introduction

*Synhimantus* (*Dispharynx*) *nasuta* (Rudolphi, 1819) (Spirurida: Acuariidae) is a nematode parasite that inhabits the gastrointestinal system of domestic and wild birds worldwide. It can be found in different organs such as the esophagus, gizzard, proventriculus and small intestine (Zhang et al., 2004; Carreno, 2008).

This nematode has a wide geographic distribution, with records of infection in North, Central and South America, Africa, Australia, Europe and Asia (Carreno, 2008). In Brazil, presence of *S.* (*D.*) *nasuta* has been reported in the domestic birds as *Gallus gallus domesticus* Linnaeus, 1758, *Meleagris gallopavo* Linnaeus, 1758, and *Columba livia* Gmelin, 1789 (Vicente et al., 1995), *Pavo cristatus* Linnaeus, 1758 (Duarte & Dórea, 1987), *Passer domesticus* Linnaeus, 1758 (Brasil & Amato, 1992); *Phasianus colchicus* Linnaeus, 1758 (Pinto et al., 2004). In wild birds from Brazil, this parasite was previously reported in *Guira guira* Gmelin, 1788, and *Crotophaga ani* Linnaeus, 1758 (Bartmann & Amato, 2009), *Paroaria capitata* Orbigny & Lafresnaye, 1837 (Mascarenhas et al., 2009), *Pitangus sulphuratus* Linnaeus, 1766 (Mendes, 2011), *Molothrus bonariensis* Gmelin, 1789 (Bernardon et al., 2016) and *Vanellus chilensis* Molina, 1782 (Silveira & Calegaro-Marques, 2016).

The aim of this study was to register the first occurrence of *S*. (*D*.) *nasuta* in some species of wild birds in Brazil. In addition, the aim was to deepen the knowledge about the morphology of this species through analysis using scanning electron microscopy analyses.

## Material and Methods

Were necropsied a total of 69 wild birds of the species *Turdus leucomelas* Vieillot, 1818 (Passeriformes: Turdidae) (N=5), *Turdus rufiventris* Vieillot, 1818 (Passeriformes: Turdidae) (N=22), *Mimus saturninus* Lichtenstein, 1823 (Passeriformes: Mimidae) (N=2), *Pitangus sulphuratus* Linnaeus, 1766 (Passeriformes: Tyrannidae) (N=20), *Megascops choliba* Vieillot, 1817 (Strigiformes: Strigidae) (N=13), *Tyto furcata* Scopoli, 1769 (Strigiformes: Tytonidae) (N=3), and *Falco sparverius* Linnaeus, 1758 (Falconiformes: Falconidae) (N=4) over a period of time between 2013 and 2017. These birds were received at Wild Animal Screening Center (CETAS) of Instituto Chico Mendes de Conservação da Biodiversidade (ICMBio), in the municipality of Juiz de Fora, after being collected in clandestine breeding and trafficking, abuse, and sales, in the Zona da Mata region in the state of Minas Gerais. The birds died in captivity were sent under freezing conditions for helminthological studies to the Laboratório de Taxonomia e Ecologia de Helmintos – Odile Bain (LATECH Odile Bain), of the Departamento de Zoologia of the Universidade Federal de Juiz de Fora (UFJF).

The parasites collected in these birds were fixed in 4% Formalin at room temperature (nematodes were dead at the time of collection) and kept in this fixative for 7 days and stored in 70ºGL ethanol (Vieira et al., 2015). For morphological and morphometric studies in the light microscope Olympus BX41 with drawing tube, the nematodes, after washing in current water, were clarified in Amann's lactophenol and mounted in temporary slides (Amato & Amato, 2010). The number of nematodes analyzed in light microscopy was selected according to the morphological condition of the parasites and the size of the infrapopulations in each host specimens. Photographs in light microscopy were taken under differential interference contrast microscopy (DIC) using an Olympus BX51 microscope coupled with an Olympus UC 30 digital camera. For scanning electron microscopy (SEM) a sample of these helminths was dehydrated in increasing series of ethanol, dried in 97% 1,1,1,3,3,3-Hexamethyldisilazane, mounted in stubs with carbon tape, gold coated, and analysed in a scanning electron microscope JEOL JSM 6390LV SEM (Vieira et al., 2015), in the Plataforma de Microscopia Eletrônica – Rudolf Barth, Instituto Oswaldo Cruz, FIOCRUZ, Rio de Janeiro.

The identification to generic level of the nematodes collected in the proventriculus was made according to Chabaud (1975). For the specific identification, studies with morphological and morphometric data of *Synhimantus* (*Dispharynx*) were consulted (Zhang et al., 2004; Dewi et al., 2006; Bartmann & Amato, 2009; Oyarzún-Ruiz et al., 2016; Hernandez-Urraca et al., 2022). The morphometric data are provided in micrometers (µm), except when another unit is informed. The prevalence, mean intensity, and mean abundance of helminth were calculated according to Bush et al. (1997). Voucher specimens have been deposited in the Coleção Helmintológica Odile Bain (CHOB), Departamento de Zoologia, Universidade Federal de Juiz de Fora, in the state of Minas Gerais, Brazil.

## Results

### Description

**Figure 1:**
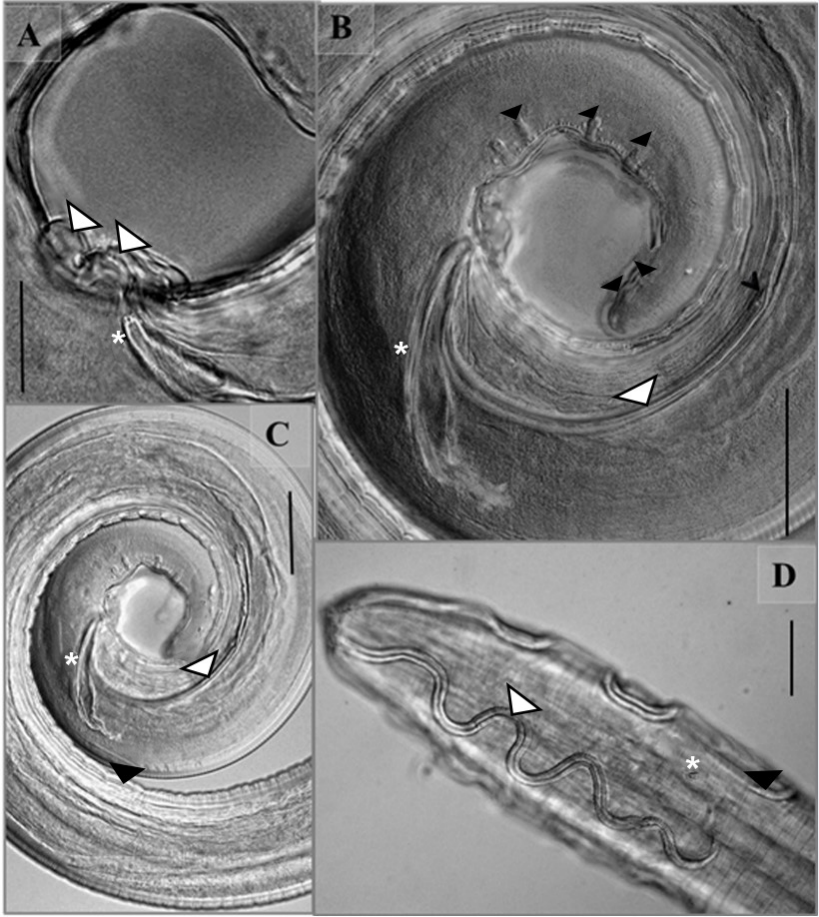
*Synhimantus* (*Dispharynx*) *nasuta* from *Pitangus sulphuratus*, at light microscope. A, Male, cuticular projections in the cloacal region (white arrow); distal end of the right spicule (asterisk); B, Male, postcloacal papillae (black arrow); right spicule short (asterisk); left spicule long (white arrow); boundary between the shaft and the lamina of the left spicule (black setae); C, Male, right spicule short (asterisk); left spicule long (white arrow); D, Male. descending cephalic cordons (white arrow); ascending cephalic cordons (black arrow); cervical papillae (asterisk). Scale: A, 90μm; B 40μm; 60μm; C 40μm; D 60μm.

**Figure 2:**
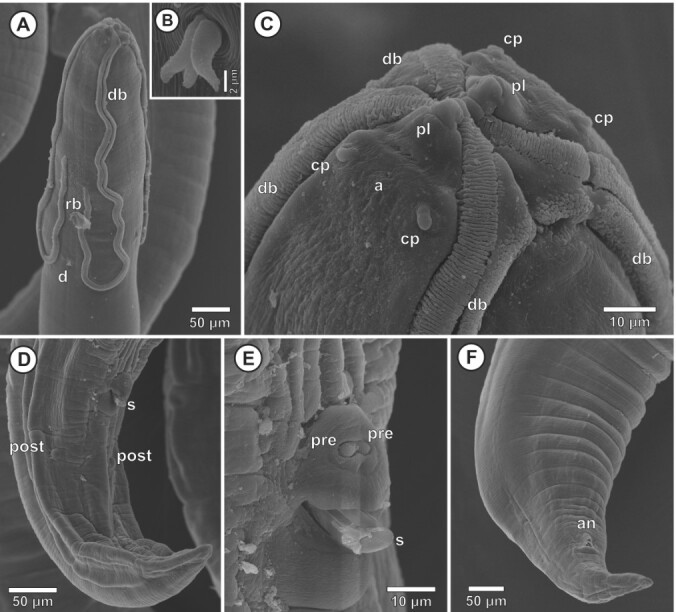
*Synhimantus* (*Dispharynx*) *nasuta* from *Megascops choliba*, at Scanning Electron Microscope (SEM). A, Male. Anterior region. Latero apical view. B, Male, deirid, lateral view. C, Male, anterior end. Apical view; D, Male. Posterior end. Latero ventral view; E. Male. Cloacal region, ventral view; F. Female. Latero ventral view (abbreviations: a – amphid, an – anus, cp - cephalic papapilla, d – deirid, db - descending branch of cord, pl – pseudo labia, post – post cloacal papilla, pre – pre cloacal papilla, rb - recurrent branch of cord, s - spicule).

*Synhimantus* (*Dispharynx*) *nasuta* (Rudolphi, 1819)

(Figures 1 and 2, Table 1)

**Table 1:** Morphometric data of males and females of *Synhimantus* (*Dispharynx*) *nasuta* in each host species, in the Microregion of Zona da Mata Mineira, Minas Gerais, Brazil.

**Hosts species (Number of analyzed hosts)**	***Turdus rufiventris* (N=22)**		***Turdus Leucomelas* (N=5)**		***Pitangus sulphuratus* (N=20)**		***Mimus Saturninus* (N=2)**		***Tyto furcata* (N=2)**		***Falco sparverius* (N=4)**		***Megascops choliba* (N=13)**	
**Prevalence**	22.7%		20%		65%		50%		33.3%		100%		69%	
	**Male (n=10)**	**Femal (n=10)**	**Male (n=5)**	**Female (n=5)**	**Male -(n=10)**	**Female (n=10)**	**Male (n=2)**	**Female (n=2)**	**Male (n=2)**	**Female (n=2)**	**Male (n=10)**	**Female (n=10)**	**Male (n=5)**	**Female (n=5)**
Total Body length (mm)	5.3–6	5–6.3	5.3–5.4	5.4–6	6.2–6.3	6.9–7.2	4.8–6	6–6.2	5.5–6.1	6–7.7	5.7–5.9	5.7–6.5	6.2–7.7	8.3–9.1
Body width	140–380	280–430	165–368	340–360	250–275	255–440	200–240	305–390	250–280	300–450	290–300	320–365	250–290	550–670
Descending branch of cordons length	335–450	345–595	380	420–505	375–385	530–590	350–370	410–470	390–420	410–530	360–400	375–590	530–400	570–760
Recurrent branch of cordons length	100–160	170–360	65–155	180–310	86–120	210–270	70–100	175–195	120–170	190–325	45–130	200–295	185–255	305–475
Deirids to anterior end	260–385	280–460	340–360	305–390	320–360	320–455	290–340	325–380	290–400	345–440	370–380	325–365	405–485	500–635
Buccal capsule length	118–130	90–170	80–120	90–115	100–140	110–165	100–115	100–145	100–130	125–150	75–120	120–135	100–130	120–170
Muscular esophagus length	400–540	570–660	440–450	510–635	270–410	670–680	350–400	545–570	360–510	490–680	405–550	415–600	440–690	460–650
Glandular esophagus length (mm)	1.1–1.5	1.3–1.6	1.3–1.4	0.8–1.3	1–1.7	1.6–2.2	1.3–2	1–1.7	1.5–1.6	1.8–1.9	1.5–1.7	1.3–1.7	1.35–1.73	1–2.1
Left spicule length	317–390	-	310–370	-	320–365	-	300–375	-	390–410	-	348–397	-	300–400	
Right spicule length	160–180	-	170–180	-	180–185	-	180–190	-	190–200	-	185–205	-	120–190	
Longitudinal striations length (mm)	1.6–1.9	-	1.65–1.8	-	1.7–2.1	-	1.6–1.7	-	1.8–2	-	1.95–2.4	-	1.79–2.6	
Longitudinal striations extending to anterior end (mm)	3.3–3.8	-	3.2–3.5	-	3.4–4.1	-	2.8–3.3	-	3.2–4.7	-	4.2–4.8	-	5.1–5.8	
Precloacal papillae	4	-	4	-	4	-	4	-	4	-	4	-	4	
Postcloacal papillae	6	-	6	-	6	-	5	-	6	-	6		5	
Vulva from posterior end (mm)	-	1.4–2	-	1–1.8	-	1.6–1.9	-	1.7–1.8	-	1.5–2	-	1.7–1.9	-	1.6–2
Tail length	230–325	160–205	240–310	150–195	390–450	185–210	340–345	195–200	360–430	220–355	320–375	255–270	270–340	150–210

General: White-colored nematodes. Robust, filiform body with transversally striated cuticle (Figure 2A, 2D, 2F). Sexual dimorphism present, with females larger than males. Two pseudolips small and conical (Figure 2C), with one small amphid in each (Figure 2C). Short buccal capsule striated transversely. Esophagus divided into short muscular and long glandular portions. Four distinct, unanastomosed, convoluted branch, beginning on the dorsal and ventral surface of the oral opening, extending to the posterior region of the muscular esophagus (Figure 2A, 2C). Nerve ring anterior to muscular esophagus. Excretory pore posterior to the nerve ring. Deirids bicuspid or tricuspid (Figure 2B), at level of excretory pore, located between recurrent branches of the cords (Figure 2A).

Males (n=10 from *F. sparverius;* n=10 from *P*. *sulphuratus*; n=10 from *T*. *rufiventris*; n=5 from *T*. *leucomelas*; n=2 from *M*. *saturninus;* n=2 from *T. furcata*; n=5 from *M. choliba*) (morphometric data of speciemens in each host are in the Table 1): Posterior region with one or two pairs of sessile papillae near the tail. Four pairs of pre-cloacal papillae and five or six post-cloacals papillae (Figure 1B). Irregular spicule (Figures 1B, 1C). Left spicule long and slender, right spicule short and thick (Figure 1B, 1C). Ventral surface of precloacal region with prominent longitudinal ridges (Figures 1B, 1C; Figures 2D, 2E). Tail bluntly rounded (Figure 1B; Figure 2D).

Females (n=10 from *F. sparverius;* n=10 from *P*. *sulphuratus*; n=10 from *T*. *rufiventris*; n=5 from *T*. *leucomelas*; n=2 from *M*. *saturninus;* n=2 from *T. furcata*; n=5 from *M. choliba*) (morphometric data of speciemens in each host are in the Table 1): Didelphic. Vulva located in the end of body. Tail short, conical, filiforme (Figure 2F). Eggs ellipsoid, thick shelled.

### Taxonomic Summary 

Hosts: *T. leucomelas* Vieillot, 1818, *T. rufiventris* Vieillot, 1818, *M. saturninus* Lichtenstein, 1823, *P. sulphuratus* Linnaeus, 1766, *M. choliba* Vieillot, 1817, *T. furcata* Scopoli, 1769, and *F. sparverius* Linnaeus, 1758.

Site of infection: Proventriculus, embedded in the mucosa or free.

Prevalences: 20% in *T. leucomelas*; 22.7% in *T. rufiventris*; 50% in *M. saturninus*; 65% in *P. sulphuratus;* 69% in *M. choliba*; 33.3% in *T. furcata*; and 100% in *F. sparverius*.

Intensity or mean intensity (range of infrapopulations): 33 specimens in *Turdus leucomelas* (one infected host); 10.2 (3-27) in 5 infected *Turdus rufiventris*; 5 specimens in *Mimus saturninus* (one infected host); 4.3 (1-19) in 13 infected *Pitangus sulphuratus,* 3.5 (2-13) in 9 infected *Megascops choliba*, 5 specimens in *Tyto furcata* (one infected host), and 37.5 (11-150) in 4infected *Falco sparverius*.

Localities: Zona da Mata Mineira (Juiz de Fora, Ubá and Cataguases, Minas Gerais, Brazil).

Voucher specimens: CHOB 130 (Host: *T. leucomelas*); CHOB 129 (Host: *T. rufiventris*); CHOB 132 (host: *M. saturninus*); CHOB 131 (host: *P. sulphuratus*); CHOB 054 (host: *M. choliba*); CHOB 133 (host: *T. furcata*); and CHOB 134 (host: *F. sparverius*).

## Discussion

The morphological and morphometric data observed in the nematodes of the present study, such as the shape of the curved body in the males, striated cuticle of considerable thickness, the mouth has two conical pseudolabia close to where the cephalic attachment structures (cephalic cords) appear, are coincident with those described by previous authors (Cram, 1927; Macko et al., 1974; Zhang et al., 2004), and make it evident that the nematodes found in the present work belong to the species *S.* (*Dispharynx*) *nasuta*.

It can be observed measurement variation related to the host species (Table1). However, this values are within the limits of variation that have been reported for *S*. (*D*) *nasuta* in several descriptive studies (Zhang et al., 2004; Dewi et al., 2006; Bartmann & Amato, 2009; Oyarzún-Ruiz et al., 2016; Hernandez-Urraca et al., 2022).

Differential interference contrast (DIC) microscopy evaluations showed cuticular projections with rounded edges in the cloacal opening of males (Figure 1A): one just before the anterior edge of the cloacal opening and the other after the posterior edge (Figure 1A). These projections were not mentioned in the previous descriptions of *S*. (*D*.) *nasuta* available in the literature (Cram, 1927; Macko et al., 1974; Zhang et al., 2004; Dewi et al., 2006; Bartmann & Amato, 2009; Gómez-Puerta et al., 2009; Oyarzún-Ruiz et al., 2016; Hernandez-Urraca et al., 2022). However, in one of the figures published by Zhang et al. (2004), there are indications of the presence of these projections, although they were not highlighted by those authors. Findings like these emphasize the need for new taxonomic studies on some nematode species, especially those that have been referred to as having low specificity. Such studies should address morphology, light and scanning electron microscopy (SEM) and molecular biology.

In the present study, some morphological particularities are showing for the first time at SEM images, for example the two conical pseudolabia with a pair of labial papillae on each side and amphids (Figure 2C), and the two morphological types of deirids, i.e. bicuspid and tricuspid (Figure 2B). The images also revealed in detail the morphology of the descending and ascending cephalic cords (Figures 2A, 2C). These details at SEM were not provided by previous authors (Rodrigues et al., 2003; Dewi et al., 2006).

The SEM images also showed the rough area of the males, a region that assists in attachment for copulation (Figure 2E) and showed post-cloacal papillae (Figure 2D) and two papillae near the cloaca (Figures 2D, 2E). These had not been reported in any previous study with species description. In the study by Dewi et al. (2006), it was possible through SEM to observe in detail the pre and post cloacal papillae in males, similar to those observed in the nematodes collected in the present study. Scanning electron microscopy has become an important technical instrument for determining the taxonomy of this group since the species of the subgenus and genus have similarities with minimal morphoanatomical divergences.

In general, the prevalences recorded in the present study varied according to the host species, as observed by Zhang et al. (2004), Oyarzún-Ruiz et al. (2016), and in the review provided by Hernandez-Urraca et al. (2022), from several hosts in different localities.

Among the host species in which *S*. (*D*.) *nasuta* was found in the present study, there have been previous records of these nematodes in *F*. *sparverius* in the United States (Taft et al., 1993) and *T*. *furcata* in Italy (Santoro et al., 2012). Therefore, it can be said that the findings of the present study constitute the first record of *S*. (*D*.) *nasuta* in *F*. *sparverius* and *T*. *furcata* in South America and, at the same time, these findings expand the host range of this parasite species worldwide, through the first records in *M*. *choliba*, *M*. *saturninus*, *T*. *leucomelas* and *T*. *rufiventris*.
